# Acute Meningoencephalitis Associated with *Borrelia miyamotoi*, Minnesota, USA

**DOI:** 10.3201/eid3007.231611

**Published:** 2024-07

**Authors:** Jeffrey M. Kubiak, Michael Klevay, Evann E. Hilt, Patricia Ferrieri

**Affiliations:** M Health Fairview University of Minnesota Medical Center, Minneapolis, Minnesota, USA (J.M. Kubiak, E.E. Hilt, P. Ferrieri);; St. Paul Infectious Disease Associates, M Health Fairview, Saint Paul, Minnesota, USA (M. Klevay)

**Keywords:** bacteria, meningoencephalitis, Borrelia miyamotoi, zoonoses, vector-borne infections, tick-borne infections, microscopy, ribosomal RNA 16S, Minnesota, United States, meningitis/encephalitis, ticks

## Abstract

*Borrelia miyamotoi* is an emerging tickborne pathogen that has been associated with central nervous system infections in immunocompromised patients, albeit infrequently. We describe a case-patient in Minnesota, USA, who had meningeal symptoms of 1 month duration. *B. miyamotoi* infection was diagnosed by Gram staining on cerebrospinal fluid and confirmed by sequencing.

*Borrelia miyamotoi* is an emerging tickborne pathogen initially discovered in Japan in 1995 but not recognized as a human pathogen until described in a case series from Russia in 2011 (*1*). The bacterium has a geographic distribution across northern Europe and Asia, as well as in the northeastern and midwestern United States; the first case of *B. miyamotoi* in Minnesota, USA, was reported in 2016 (*2*). *B. miyamotoi* is spread by many species of the deer tick, including *Ixodes scapularis* in North America and *I. ricinus* in Europe and Asia. *B. miyamotoi* is classified within the relapsing-fever group of spirochetes; infection typically manifests as a nonspecific febrile illness with headaches, chills, myalgia, and arthralgia and is likely underdiagnosed because of its rarity and lack of specific symptoms (*3*,*4*). 

*B. miyamotoi* has been associated with cases of neuroborreliosis in which spirochetes proliferate in the cerebrospinal fluid (CSF) to cause disease ranging from acute meningitis symptoms to prolonged encephalitis with altered mentation and cranial nerve deficits (*5*–*8*). Here, we report a case of acute meningoencephalitis associated with *B. miyamotoi* in the midwestern United States that was diagnosed on CSF Gram stain and confirmed by sequencing. 

## The Study

A 68-year-old man with immunosuppression from rituximab, in remission from mantle cell lymphoma involving the right eye, sought treatment at an emergency department in Minnesota in late summer. He had a 5-week history of headaches of increasing severity, worsened by positional changes and physical activity, as well as double vision, hearing difficulties, and some altered mentation. The patient was afebrile and had no nuchal rigidity. Cranial nerves exhibited no dysfunction other than subjective hearing loss, and physical examination results were otherwise unremarkable. He was an avid outdoorsman who had hiked frequently in the woodlands of Minnesota and Wisconsin over the months before seeking treatment, and he recalled identifying an engorged tick among several tick bites on his body during this time. He reported no travel history outside of the midwestern United States. The patient had been treated ≈1 year earlier with doxycycline for clinically diagnosed Lyme disease with fever and erythema migrans, but serologic *B. burgdorferi* testing at the time was negative. 

We performed a magnetic resonance imaging scan of the brain and orbits with and without contrast, which showed bilateral abnormal cranial nerve enhancement of the oculomotor, trigeminal, facial, and vestibulocochlear nerves compared with a magnetic resonance image from 2 months earlier. Results of a lumbar puncture included a neutrophil-predominant pleocytosis (177 leukocytes/μL, 73% neutrophils). CSF flow cytometry results were unremarkable. A Biofire FilmArray (bioMérieux, https://www.biomerieux.com) meningitis/encephalitis multiplex PCR panel was negative. The patient had an elevated C-reactive protein (19.5 mg/L). Serologic results for *B. burgdorferi* in both the blood and CSF samples were negative, as were results of antitreponemal testing. Blood culture results were negative, and routine laboratory values were otherwise unremarkable. 

A direct CSF Gram stain was performed in the University of Minnesota Medical Center Infectious Diseases Diagnostic Laboratory (Minneapolis, MN, USA) (Figure). The CSF specimen showed numerous gram-negative bacteria that measured 10–20 μm long with a tight coil, consistent with spirochetes. The CSF specimen was sent to the University of Washington Molecular Diagnosis Microbiology Section (Seattle, WA, USA) for bacterial 16S ribosomal RNA (rRNA) sequencing and fungal 28S rRNA sequencing.

**Figure:**
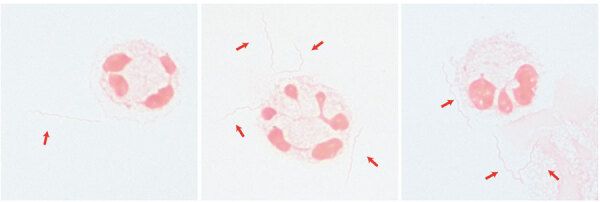
Spirochetes (red arrows) visualized on Gram stain in a cerebrospinal fluid sample from a 68-year-old man with immunosuppression from rituximab, Minnesota, USA. Visualization was done after concentration using cytospin (original magnification ×1,000 with oil immersion). The spirochetes were later identified as *Borrelia miyamotoi* by 16S ribosomal sequencing.

The patient was admitted and treated with 2 g intravenous ceftriaxone daily for presumed neuroborreliosis, pending identification of the organism. After administering the initial antimicrobial treatment, we sent a plasma sample for a tickborne relapsing fever PCR panel, which broadly detects *Borrelia* species including *B. hermsii, B. parkeri, B. turicatae*, and *B. miyamotoi*; the test result was negative. Within 24 hours, the patient’s condition improved on antimicrobial therapy, and on day 3 of hospitalization, we discharged him; he reported no headaches and some improvement in his cognition, hearing, and vision. Six days after we discharged the patient from the hospital, his 16S ribosomal sequencing result returned positive results for *B. miyamotoi*. We administered daily outpatient infusion therapy with ceftriaxone for 4 weeks, during which time he reported mild residual subjective memory loss, hearing loss, and loss of visual acuity from baseline, although double vision resolved. We advised him on appropriate tick-prevention methods because his history of outdoor activity indicated increased risk for future tick bites. 

## Conclusions

In cases of *B. miyamotoi* with neurologic manifestations, clinical symptoms have ranged from acute meningeal symptoms, including headache, vomiting, and dizziness (*5*,*9*), to prolonged neurologic disease, in some cases involving several months of progressive mental decline, confusion, gait instability, and hearing difficulty (*6*,*7*). Typically, in acute meningoencephalitis cases, spirochetes cannot be visualized in direct CSF specimen Gram or Giemsa stains (*5*). Diagnosis in those cases has relied on either broad-based 16S sequencing of bacteria rRNA, targeted PCR identification of the GlpQ or flagellin genes, darkfield microscopy, or acridine orange stain (*5*,*6*,*9*,*10*). In contrast, in 1 case, spirochetes were visualized on direct specimen Giemsa and Gram stains in the presence of prolonged neuroborreliosis of ≈4 months duration (*7*). Although literature on the subject remains lacking because of the relative rarity of this disease manifestation, those cases suggest that, in immunocompromised patients, spirochetes may proliferate in CSF over time, leading to an increased likelihood of detecting spirochetes on direct specimen examination in patients with prolonged symptoms. It is notable that DNA from *B. miyamotoi* was not detected in our patient’s plasma, suggesting a neurologic tropism that enabled continued spirochetal proliferation even after the organisms were cleared from the patient’s bloodstream. 

This patient had abnormal MRI findings, including abnormal cranial nerve enhancement of the oculomotor, trigeminal, facial, and vestibulocochlear cranial nerves. Those findings were initially worrisome for leptomeningeal progression of the patient’s ocular lymphoma, but flow cytometry of his CSF did not demonstrate abnormal proliferation of B cells. Although previous case reports have shown no abnormalities on cranial MRI (*5*–*7*), given this patient’s constellation of symptoms, we believe that these MRI findings were attributable to this infection and the proliferation of spirochetes in the CSF.

Differential diagnosis of spirochetes visualized on CSF Gram stain is limited. We found 1 other published case report with this finding, also for *B. miyamotoi* (*7*). Although other spirochetes, such as *B. burgdorferi* and *Treponema pallidum*, are known to cause neurologic manifestations ranging from acute meningoencephalitis to chronic encephalitis, those organisms are not visualized with Gram stain; diagnoses require darkfield microscopy, PCR, or serologic evidence of disease (*10*–*13*). We posit that finding spirochetes on CSF Gram stain is highly suggestive of *B. miyamotoi*, and patients should be empirically treated for neuroborreliosis pending definitive identification, particularly patients active in geographic areas with known tick exposure who have rituximab-caused immunosuppression. 

Of note, most reported cases of neuroborreliosis caused by *B. miyamotoi* have been found in older patients on rituximab therapy with B-cell depletion, as in this case-patient, although there are rare reports of neuroborreliosis in immunocompetent persons (*9*). The adaptive immune system is considered key in controlling *B. miyamotoi* infection; *B. miyamotoi* activates dendritic cells, phagocytizing the bacteria and stimulating release of several cytokines (*14*). Furthermore, studies in related species, such as *B. hermsii*, have shown that clearance of spirochetes is mediated by antibodies (*15*). Further research into the pathogenesis of *B. miyamotoi* meningoencephalitis, with an emphasis on the regulators of proliferation of these organisms in CSF and disease progression in immunocompromised patients, are certainly warranted. Greater awareness of this manifestation of *B. miyamotoi* disease is needed to increased diagnostic accuracy and antimicrobial treatment to enable improved patient outcomes. 

## References

[1] Platonov AE, Karan LS, Kolyasnikova NM, Makhneva NA, Toporkova MG, Maleev VV, et al. Humans infected with relapsing fever spirochete *Borrelia miyamotoi*, Russia. Emerg Infect Dis. 2011;17:1816–23. 10.3201/eid1710.101474 22000350 PMC3310649

[2] Minnesota Department of Health. About *Borrelia miyamotoi* disease [cited 2023 Nov 21]. https://www.health.state.mn.us/diseases/bmiyamotoi/basics.html

[3] Cleveland DW, Anderson CC, Brissette CA. *Borrelia miyamotoi*: a comprehensive review. Pathogens. 2023;12:267. 10.3390/pathogens12020267 36839539 PMC9967256

[4] McCormick DW, Brown CM, Bjork J, Cervantes K, Esponda-Morrison B, Garrett J, et al. Characteristics of hard tick relapsing fever caused by *Borrelia miyamotoi*, United States, 2013–2019. Emerg Infect Dis. 2023;29:1719–29. 10.3201/eid2909.221912 37610298 PMC10461660

[5] Boden K, Lobenstein S, Hermann B, Margos G, Fingerle V. *Borrelia miyamotoi*–associated neuroborreliosis in immunocompromised person. Emerg Infect Dis. 2016;22:1617–20. 10.3201/eid2209.152034 27533748 PMC4994329

[6] Hovius JWR, de Wever B, Sohne M, Brouwer MC, Coumou J, Wagemakers A, et al. A case of meningoencephalitis by the relapsing fever spirochaete *Borrelia miyamotoi* in Europe. Lancet. 2013;382:658. 10.1016/S0140-6736(13)61644-X 23953389 PMC3987849

[7] Gugliotta JL, Goethert HK, Berardi VP, Telford SR III. Meningoencephalitis from *Borrelia miyamotoi* in an immunocompromised patient. N Engl J Med. 2013;368:240–5. 10.1056/NEJMoa1209039 23323900 PMC4018741

[8] Kubiak K, Szczotko M, Dmitryjuk M. *Borrelia miyamotoi*—an emerging human tick-borne pathogen in Europe. Microorganisms. 2021;9:154. 10.3390/microorganisms9010154 33445492 PMC7827671

[9] Henningsson AJ, Asgeirsson H, Hammas B, Karlsson E, Parke Å, Hoornstra D, et al. Two cases of *Borrelia miyamotoi* meningitis, Sweden, 2018. Emerg Infect Dis. 2019;25:1965–8. 10.3201/eid2510.190416 31538916 PMC6759261

[10] Gyllemark P, Wilhelmsson P, Elm C, Hoornstra D, Hovius JW, Johansson M, et al. Are other tick-borne infections overlooked in patients investigated for Lyme neuroborreliosis? A large retrospective study from South-eastern Sweden. Ticks Tick Borne Dis. 2021;12:101759. 10.1016/j.ttbdis.2021.101759 34161869

[11] Cutler SJ. Relapsing fever *Borreliae.* Clin Lab Med. 2015;35:847–65. 10.1016/j.cll.2015.07.001 26593261

[12] Madison-Antenucci S, Kramer LD, Gebhardt LL, Kauffman E. Emerging tick-borne diseases. Clin Microbiol Rev. 2020;33:e00083–18. 10.1128/CMR.00083-18 31896541 PMC6941843

[13] Ramchandani MS, Cannon CA, Marra CM. Syphilis: a modern resurgence. Infect Dis Clin North Am. 2023;37:195–222. 10.1016/j.idc.2023.02.006 37005164

[14] Mason LMK, Koetsveld J, Trentelman JJA, Kaptein TM, Hoornstra D, Wagemakers A, et al. *Borrelia miyamotoi* activates human dendritic cells and elicits T cell responses. J Immunol. 2020;204:386–93. 10.4049/jimmunol.1801589 31818980

[15] Crowder CD, Ghalyanchi Langeroudi A, Shojaee Estabragh A, Lewis ERG, Marcsisin RA, Barbour AG. Pathogen and host response dynamics in a mouse model of *Borrelia hermsii* relapsing fever. Vet Sci. 2016;3:19. 10.3390/vetsci3030019 29056727 PMC5606581

